# Sulfasalazine induces mitochondrial dysfunction and renal injury

**DOI:** 10.1080/0886022X.2017.1399908

**Published:** 2017-12-07

**Authors:** Hossein Niknahad, Reza Heidari, Roya Mohammadzadeh, Mohammad Mehdi Ommati, Forouzan Khodaei, Negar Azarpira, Narges Abdoli, Mahdi Zarei, Behnam Asadi, Maryam Rasti, Babak Shirazi Yeganeh, Vahid Taheri, Arastoo Saeedi, Asma Najibi

**Affiliations:** a Pharmaceutical Sciences Research Center, Shiraz University of Medical Sciences, Shiraz, Iran;; b Department of Pharmacology and Toxicology, Faculty of Pharmacy, Shiraz University of Medical Sciences, Shiraz, Iran;; c Department of Animal Sciences, School of Agriculture, Shiraz University, Shiraz, Iran;; d Transplant Research Center, Shiraz University of Medical Sciences, Shiraz, Iran;; e Food and Drug Organization, Ministry of Health, Tehran, Iran;; f Department of Pathology, School of Medicine, Shiraz University of Medical Sciences, Shiraz, Iran

**Keywords:** Anti-rheumatoid drugs, drug metabolite, energy crisis, mitochondria, nephrotoxicity, oxidative stress, renal injury, sulphasalazine

## Abstract

Sulfasalazine is a commonly used drug for the treatment of rheumatoid arthritis and inflammatory bowel disease. There are several cases of renal injury encompass sulfasalazine administration in humans. The mechanism of sulfasalazine adverse effects toward kidneys is obscure. Oxidative stress and its consequences seem to play a role in the sulfasalazine-induced renal injury. The current investigation was designed to investigate the effect of sulfasalazine on kidney mitochondria. Rats received sulfasalazine (400 and 600 mg/kg/day, oral) for 14 consecutive days. Afterward, kidney mitochondria were isolated and assessed. Sulfasalazine-induced renal injury was biochemically evident by the increase in serum blood urea nitrogen (BUN), gamma-glutamyl transferase (γ-GT), and creatinine (Cr). Histopathological presentations of the kidney in sulfasalazine-treated animals revealed by interstitial inflammation, tubular atrophy, and tissue necrosis. Markers of oxidative stress including an increase in reactive oxygen species (ROS) and lipid peroxidation (LPO), a defect in tissue antioxidant capacity, and glutathione (GSH) depletion were also detected in the kidney of sulfasalazine-treated groups. Decreased mitochondrial succinate dehydrogenase activity (SDA), mitochondrial depolarization, mitochondrial GSH depletion, increase in mitochondrial ROS, LPO, and mitochondrial swelling were also evident in sulfasalazine-treated groups. Current data suggested that oxidative stress and mitochondrial injury might be involved in the mechanism of sulfasalazine-induced renal injury.

## Introduction

Sulfasalazine is widely used in the management of inflammatory bowel diseases and rheumatoid arthritis in humans [1]. Sulfasalazine is metabolized to sulfapyridine and mesalamine (mesalazine) by bacterial azoreductase enzyme in the colon. It is expected that approximately 10–30% of sulfasalazine is absorbed unchanged to the systemic circulation [1]. Sulfapyridine is completely absorbed. On the other hand, 30% of formed mesalazine reaches circulation, and the rest is excreted in feces [1].

Although sulfasalazine is generally considered as a safe medication [2], several cases of renal injury have been reported with sulfasalazine administration [3–7]. There is no precise mechanism(s) for sulfasalazine-induced renal injury. Some investigations mentioned the role of oxidative stress in this complication [8–10].

Mitochondria are recognized as the producers of the majority of energy need for cellular normal activity. Many xenobiotics are capable of inducing mitochondrial injury. Among these, several pharmaceuticals are reported to affect mitochondrial function [11,12]. Energy metabolism disorders can affect practically any organ. On the other hand, high and constant dependence of kidney proximal tubular cells on energy mentions the importance of mitochondria in this organ [13–15]. Oxidative stress could act as a cause or a consequence of mitochondrial dysfunction [16]. Hence, sulfasalazine-induced oxidative stress might lead to mitochondrial injury and vice versa.

The current investigation was designed to evaluate the role of mitochondrial dysfunction in sulfasalazine-induced kidney injury. In this context, sulfasalazine was administered to rats and different serum and tissue biomarkers along with several mitochondrial indices have been evaluated.

## Materials and methods

### Chemicals

Dichlorofluorescein diacetate (DCFH-DA), malondialdehyde (MDA), 3-[4,5-dimethylthiazol-2-yl]-2,5-diphenyltetrazolium bromide (MTT), trichloroacetic acid (TCA), sulfasalazine, Coomassie brilliant blue, 2,4,6-tripyridyl-s-triazine (TPTZ), glutathione (GSH), sucrose, d-mannitol,3-(N-morpholino) propane sulfonic acid (MOPS), bovine serum albumin (BSA), rhodamine 123 (Rh-123), ferric chloride hexahydrate (FeCl_3_.6H_2_O), dithiothreitol (DTT), 6-hydroxy-2,5,7,8-tetramethylchroman-2-carboxylic acid (Trolox), sodium succinate, and thiobarbituric acid (TBA) were purchased from Sigma (Sigma-Aldrich, St. Louis, MO). Kits for evaluating biomarkers of renal injury were obtained from Pars Azmun (Tehran, Iran). Ethylenediaminetetraacetic acid (EDTA), 5,5-bis-dithio-nitro benzoic acid (DTNB), 4-(2-hydroxyethyl)-1-piperazineethanesulfonic acid (HEPES), orthophosphoric acid (OPA), n-butanol, and 2-amino-2-hydroxymethyl-propane-1,3-diol (Tris) were obtained from Merck (Darmstadt, Germany). All salts used for making buffer solutions were of analytical grade and obtained from Merck (Darmstadt, Germany).

### Animals

Male Sprague Dawley rats (*n* = 24) (Animal Breeding Center, Shiraz University of Medical Sciences, Shiraz, Iran) weighing 200–250 g were housed in an environmental temperature of 23 ± 1 °C with a 40% of relative humidity and a 12 L: 12 D photoschedule. Rats had free access to tap water and a normal chow diet. All procedures involving the rats were in accordance with the guidance for care and use of experimental animals and were approved by a local ethic committee in Shiraz University of Medical Sciences, Shiraz, Iran (94–01-36–9606).

### Experimental setup

Animals were randomly allotted into three groups (*n* = 8). Rats were treated as follows: (1) control (vehicle-treated), (2) sulfasalazine (400 mg/kg/day, oral); and (3) sulfasalazine (600 mg/kg/day, oral). It has been previously reported that a dose of 600 mg/kg/day of sulfasalazine for 14 consecutive days caused marked renal injury in rats [8,10]. At the end of experiments, animals were anesthetized (ketamine/xylazine; 100/10 mg/kg, i.p.) and their blood and kidney samples were collected.

### Serum biochemistry and kidney histopathology

Standard kits and a Mindray BS-200^®^ auto analyzer were employed to assess serum gamma-glutamyl transpeptidase (γ-GT), cratinine (Cr), and blood urea nitrogen (BUN) [17]. For histopathological assessments, samples of kidney tissue were fixed in a buffered formalin solution (0.4% of sodium phosphate monobasic, NaH_2_PO_4_, 0.64% of sodium phosphate dibasic, Na_2_HPO_4_, and 10% of formaldehyde in distilled water; pH = 7.4). Finally, paraffin-embedded sections of tissue were prepared and stained with hematoxylin and eosin (H&E) before light microscope viewing [18].

### Kidney reactive oxygen species (ROS)

Kidney samples (200 mg) were homogenized (Heidolph homogenizer, Germany) in ice-cooled Tris-HCl buffer (40 mM, pH = 7.4, 4 °C) (1:10 w/v). Then, 100 µL of tissue homogenate was mixed with 1 mL of Tris-HCl buffer (40 mM, pH = 7.4) and 5 µL of 2′,7′-DCFH-DA (final concentration of 10 µM). The mixture was incubated for 30 min at 37 °C (Gyromax^™^ incubator shaker). Finally, the fluorescence intensity of the samples was assessed using a FLUOstar Omega^®^ multifunctional microplate reader (λ_excitation_ =  485 nm and λ_emission_ =  525 nm) [17,19].

### Lipid peroxidation in kidney tissue

Thiobarbituric acid reactive substances (TBARS) were assessed in the kidney as an index of lipid peroxidation. Briefly, 500 µL of kidney tissue homogenate (10% w/v in KCl, 1.15%, 4 °C) was added to a reaction mixture consisted of 1 mL of TBA (0.375%, w/v) and 3 mL of phosphoric acid (1% w/v, pH = 2). Samples were mixed and heated in boiling water (100 °C, 45 min). Afterward, 2 mL of n-butanol was added and vigorously mixed. Finally, samples were centrifuged (3000 ×*g* for 5 min) and the absorbance of developed color in n-butanol phase was measured at 532 nm using an Ultrospec 2000^®^ UV spectrophotometer (Uppsala, Sweden) [20].

### Kidney glutathione content

The kidney glutathione (GSH) content was assessed by the method described by Sedlak et al. [21]. Briefly, kidney tissue samples (200 mg) were homogenized (Heidolph homogenizer, Germany) in 8 mL of ice-cooled EDTA solution (20 mM, 4 °C). Then, 5 ml of the prepared homogenate was mixed with 4 mL of distilled water (4 °C) and 1 mL of TCA (50% w/v). Tubes were centrifuged (10,000 ×*g*, 4 °C, 25 min), then 2 mL of the supernatant was mixed with 4 mL of ice-cooled Tris-HCl buffer (pH = 8.9, 4 °C) and 100 µL of Ellman’s reagent (DTNB, 0.01 M in methanol). The absorbance of the developed yellow color was measured at 412 nm using an Ultrospec 2000^®^ UV spectrophotometer (Uppsala, Sweden) [22].

### Ferric reducing antioxidant power (FRAP) of kidney tissue

FRAP assay measures the change in absorbance at 593 nm due to the formation of a blue colored Fe^2+^-tripyridyltriazine compound from the colorless oxidized Fe^3+^ form by the action of electron-donating antioxidants [23]. Briefly, the working FRAP reagent was prepared by mixing 10 volumes of 300 mmol/L acetate buffer, pH = 3.6, with 1 volume TPTZ (10 mmol/L in 40 mmol/L hydrochloric acid) and with 1 volume of ferric chloride (20 mmol/L). All solutions were used on the day of preparation. Kidney tissue was homogenized in ice-cooled Tris buffer (0.25 M, containing 0.2 M sucrose and 5 mM DTT, pH = 7.4). Then, 50 µL of tissue homogenate and 150 µL of deionized water were added to 1.5 mL of the FRAP working solution. The reaction mixture was incubated at 37 °C for 5 min. Finally, samples were centrifuged (13,000 ×*g*, 1 min) and the absorbance of developed color was measured at 595 nm by an Ultrospec 2000^®^ spectrophotometer (Uppsala, Sweden) [24].

### Kidney mitochondria isolation

Rats kidneys were rapidly removed, washed, and minced in an ice-cold buffer medium (75 mM d-mannitol, 225 mM sucrose, 0.5 mM EGTA, 2 mM HEPES, 0.1% of essentially fatty acid-free BSA, pH = 7.4). Then, minced tissues were transported into a fresh buffer in a proportion of 5 mL/g of kidney and homogenized. Mitochondria were isolated by differential centrifugation of the kidney homogenate [25,26]. First, unbroken cells and nuclei were pelleted at 1000 ×*g* for 10 min at 4 °C; second, the supernatant was centrifuged at 15,000 ×*g* for 10 min at 4 °C to pellet the mitochondria. This step was repeated three times using fresh buffer medium. Final mitochondrial pellets were suspended in Tris buffer containing 320 mM sucrose, 1 mM EDTA, and 10 mM Tris-HCl, pH = 7.4, except for the mitochondrial samples used to assess ROS production, mitochondrial depolarization, and mitochondrial swelling, which were suspended in respiration buffer (320 mM sucrose,10 mM Tris, 20 mM Mops, 50 μM EGTA, 0.5 mM MgCl_2_, 0.1 mM KH_2_PO_4_, and 5 mM sodium succinate), MMP assay buffer (220 mM sucrose, 68 mM d-mannitol, 10 mM KCl, 5 mM KH_2_PO_4_, 2 mM MgCl_2_, 50 μM EGTA, and 10 mM HEPES) and swelling buffer (125 mM sucrose, 65 mM KCl, 10 mM HEPES, pH = 7.2) [25,27]. Samples protein concentrations were determined by the Bradford method [26].

### Mitochondrial MTT assay

The MTT assay was applied as colorimetric method for determination of mitochondrial succinate dehydrogenase activity (SDA); as previously described by Mosmann et al. [28]. Briefly, a mitochondrial suspension (500 mg protein/mL) was incubated with 0.4% of MTT at 37 °C for 30 min. The product of purple formazan crystals was dissolved in 1 mL dimethyl sulfoxide (DMSO) and the optical density (OD) at 570 nm was measured with an EPOCH plate reader (Bio-Tek^®^ Instruments, Highland Park, IL).

### Mitochondrial depolarization

Mitochondrial uptake of the cationic fluorescent dye, Rh-123, has been used for the estimation of mitochondrial depolarization [27,29–31]. Rh-123 accumulates in intact mitochondria by facilitated diffusion. When the mitochondrion is damaged and depolarized, there is no facilitated diffusion and the amount of Rh-123 in the supernatant is increased. In the current investigation, the mitochondrial fractions (0.5 mg protein/mL) were incubated with 10 µM of Rh-123 in the MMP assay buffer for 30 min. Afterward, samples were centrifuged (15,000 ×*g*, 1 min, 4 °C) and the fluorescence intensity of the supernatant was monitored using a FLUOstar Omega^®^ multifunctional microplate reader (λ_excitation_ = 485 nm and λ_emission_ = 525 nm) [27,32,33].

### ROS in isolated kidney mitochondria

The 2′,7′-DCFH-DA was used as a fluorescent probe to assess the mitochondrial ROS measurement in the kidney. Briefly, isolated kidney mitochondria were placed in respiration buffer containing 320 mM sucrose, 10 mM Tris-HCl, 20 mM MOPS, 50 μM EGTA, 0.5 mM MgCl_2_, 0.1 mM KH_2_PO_4_, and 5 mM sodium succinate, pH = 7.4. Then, 10 µL of DCFH-DA was added (final concentration, 10 μM) to medium and then incubated for 30 min. Then, the fluorescence intensity of DCF in samples was measured using a FLUOstar Omega^®^ multifunctional microplate reader (λ_excitation_ =  485 nm and λ_emission_ =  525 nm) [27].

### Mitochondrial swelling

Analysis of mitochondrial swelling after the isolated mitochondria (0.5 mg protein/mL) was estimated through changes in light scattering as monitored spectrophotometrically at 540 nm (30 °C) as previously described [27,31]. Briefly, isolated mitochondria were suspended in swelling buffer containing 70 mM sucrose, 230 mM d-mannitol, and 3 mM HEPES, pH = 7.2 [25]. The absorbance was measured at 540 nm during 70 min of incubation using an EPOCH plate reader (Bio-Tek Instruments^®^). A decrease in absorbance indicates an increase in mitochondrial swelling [27].

### Mitochondrial glutathione (GSH) content

Mitochondrial GSH level was determined with a method using Ellman’s reagent [21]. Isolated kidney mitochondria were suspended in phosphate buffer (pH = 7.4) and treated with TCA (10% w/v) to extract mitochondrial glutathione. The mixture was centrifuged (13,000 ×*g*, 4 °C for 1 min) to remove denatured proteins. The intensity of produced yellow color in the samples was recorded at 412 nm with an ultraviolet spectrophotometer (Pharmacia Biotech 2000^®^, Uppsala, Sweden) [27,34].

### Lipid peroxidation in kidney mitochondria

Thiobarbituric acid-reactive substances (TBARS) test was used for lipid peroxidation assay in isolated kidney mitochondria [27]. As previously mentioned, sucrose interferes with the TBARS assay [27,35]. Hence, isolated mitochondria were washed once (to remove sucrose) in ice-cooled MOPS-KCl buffer (50 mM MOPS, 100 mM KCl, pH = 7.4), and resuspended in MOPS–KCl buffer [27]. Afterward, the mitochondrial suspension was mixed with twice its volume of 15% of TCA, 0.375% of thiobarbituric acid (TBA), 0.24 N HCl and 0.5 mM of Trolox, and heated for 15 min at 100 °C [27]. After centrifugation (15,000 ×*g*, 10 min), the absorbance of the supernatant at 532 nm was recorded with an EPOCH plate reader (Bio-Tek^®^ Instruments) [27].

### Statistical analysis

Data are given as the mean ± SD. Data comparison was performed by the one-way analysis of variance (ANOVA) with Tukey’s multiple comparison test as a *post hoc*. Differences were considered statistically significant when *p* < .05.

## Results

**Figure 1.**
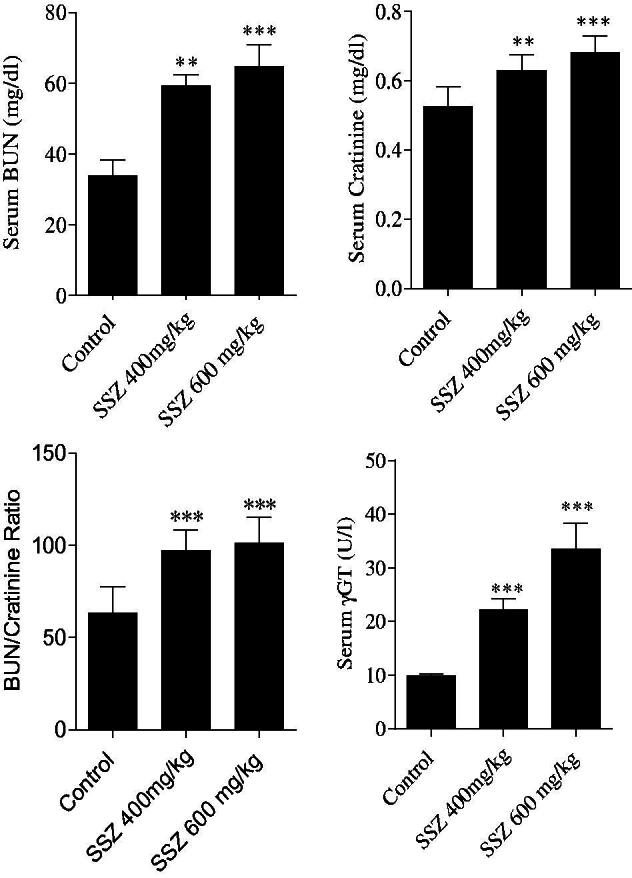
Serum biochemistry of kidney injury biomarkers in sulfasalazine-treated animals. SSZ: sulfasalazine. Data are expressed as mean ± SD (*n* = 8). Asterisks indicate significantly different as compared with control group (**p < .01 and ***p < .001).

**Figure 2.**
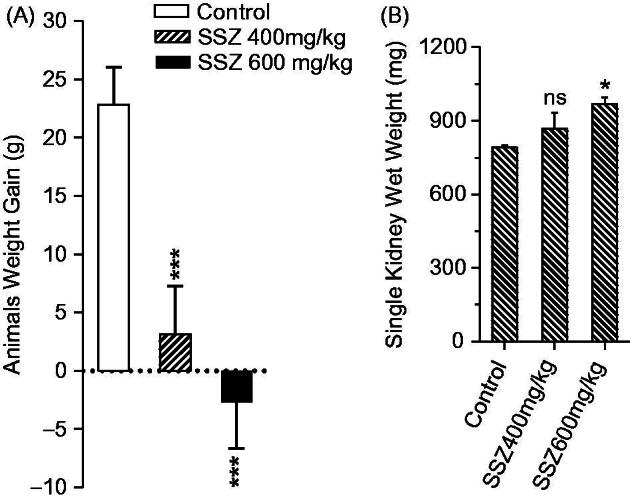
Animals weight gain (A) and kidney weight (B) were assessed after 14 d of sulfasalazine administration. SSZ: sulfasalazine. Data are given as mean ± SD (*n* = 8). Asterisks indicate significantly different as compared with control animals (*p < .05 and ***p < .001). Superscript “ns” indicates not significant as compared to control group (*p* > .05).

The sulfasalazine-induced renal injury was biochemically evident by the increase in serum BUN, gamma-glutamyl transpeptidase (γ-GT), and creatinine (Cr) in drug-treated animals (Figure 1). Moreover, it was found that animals’ weight was lower in sulfasalazine-treated groups (Figure 2) and an increment in kidney weight was detected in sulfasalazine-treated rats as compared with the control group (Figure 2).

**Figure 3.**
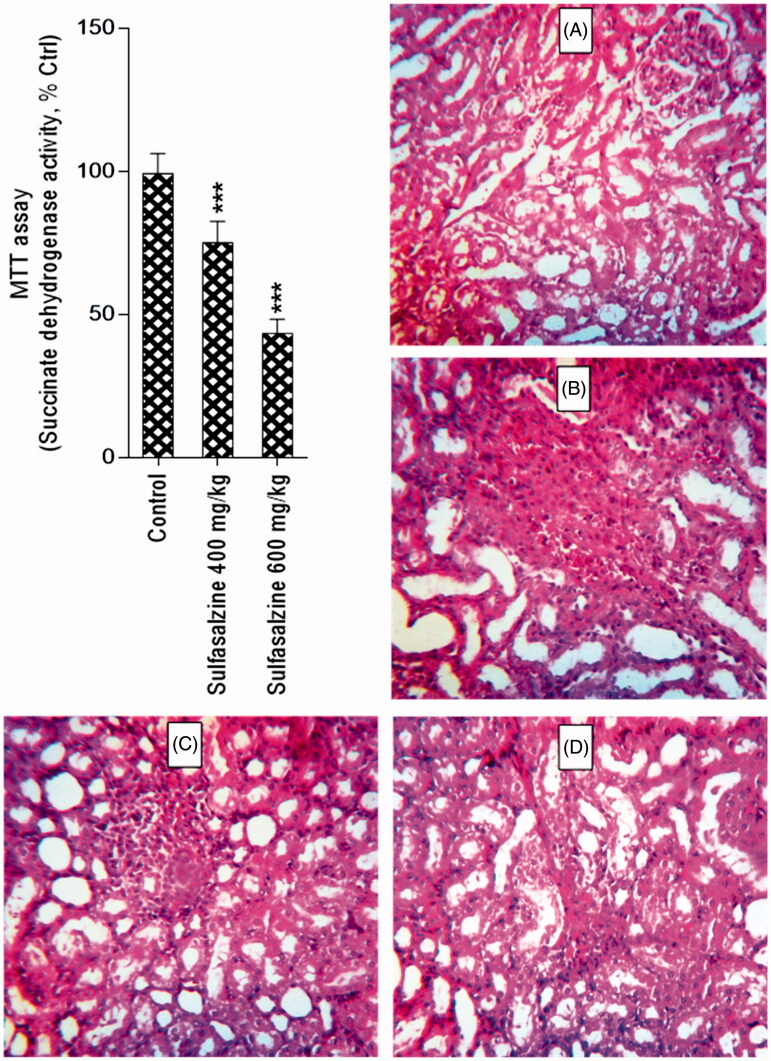
Kidney mitochondrial succinate dehydrogenase activity (SDA) (MTT assay) and photomicrographs of kidney histopathological changes in sulfasalazine-treated animals. MTT test revealed a significant decrease in mitochondrial SDA in the kidney of sulfasalazine-treated animals (mean ± SD, *n* = 8, and ***p < .001). Kidney photomicrographs showed tubular atrophy, necrosis, and interstitial inflammation in sulfasalazine-treated animals (B, C, and D) in comparison with control group (A). A: control (vehicle-treated), B: sulfasalazine 400 mg/kg/day); C and D: sulfasalazine 600 mg/kg/day).

The level of oxidative stress markers in kidney was significantly changed in sulfasalazine-treated animals (Table 1). Sulfasalazine (400 and 600 mg/kg) caused an increase in kidney ROS formation and lipid peroxidation (Table 1). Furthermore, renal GSH reservoirs were depleted and tissue antioxidant capacity defected in drug-treated rats in comparison with control group (Table 1). Histopathological presentations of the kidney in sulfasalazine-treated animals revealed by interstitial inflammation, tubular atrophy, vascular congestion, and tissue necrosis (Figure 3 and Table 2).

**Table 1.** Kidney tissue ROS formation, lipid peroxidation, total antioxidant capacity, and glutathione content.

Treatment	ROS formation (fluorescent intensity, FI)	Lipid peroxidation (nmol of TBARS/mg kidney tissue)	GSH (µmol/mg kidney tissue)	Total antioxidant capacity (µmol of vitamin C equivalent)
Control	75432 ± 5276	1.71 ± 0.82	71.55 ± 6.22	89.22 ± 11.23
SSZ 400 (mg/kg)	154209 ± 11231*	3.44 ± 0.56*	56.33 ± 3.22*	36.49 ± 6.22*
SSZ 600 (mg/kg)	186532 ± 8638*	2.66 ± 0.61*	42.35 ± 6.27*	41.22 ± 3.44*

Note: Data are shown as mean ± SD (*n* = 8). SSZ: sulfasalazine.

Asterisk(*) indicates significantly different as compared to control group (*p* < .001).

**Table 2.** Renal injury score in sulfasalazine-treated rats.

Treatment	Focal necrosis	Tubular atrophy	Interstitial inflammation	Vascular congestion
Control	–	–	–	–
SSZ 400 (mg/kg)	+	+	+	+
SSZ 600 (mg/kg)	+	+	++	++

SSZ: sulfasalazine.

**Figure 4.**
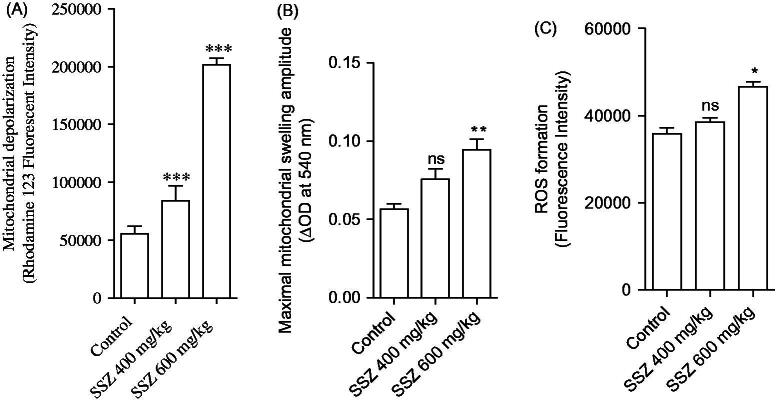
Mitochondrial depolarization (A), swelling (B), and ROS formation (C) in the kidney of sulfasalazine-treated animals. SSZ: sulfasalazine. Data are given as mean ± SD (*n* = 8). Asterisks indicate significantly different as compared with control group (*p < .05, **p < .01, and ***p < .001). Superscript “ns” indicates not significant as compared to control group (*p* > .05).

**Figure 5.**
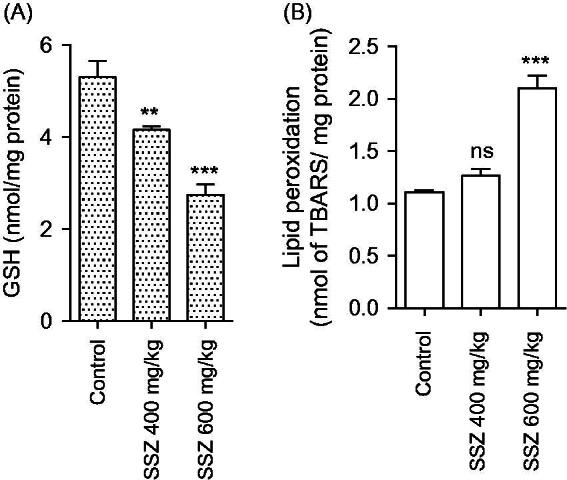
Mitochondrial glutathione content (A) and lipid peroxidation (B) in the kidney of sulfasalazine-treated animals. SSZ: sulfasalazine. Data are given as mean ± SD (*n* = 8). Superscript “ns” indicates not significant as compared to control. Asterisks indicate significantly different as compared to control (**p < .01 and ***p < .001).

Mitochondria isolated from sulfasalazine-treated animals showed marked decrease in SDA (MTT assay) (Figure 3). Further assessment of kidney mitochondria derived from sulfasalazine-treated rats revealed a marked increase in mitochondrial swelling, mitochondrial depolarization, and an increase in mitochondrial ROS level (Figure 4). It was also detected that renal mitochondrial glutathione stores were decreased in sulfasalazine-treated animals (Figure 5). sulfasalazine administration (600 mg/kg) also increased mitochondrial lipid peroxidation in rat kidney (Figure 5).

## Discussion

Sulfasalazine is widely administered in a variety of inflammatory-based diseases. On the other hand, several cases of sulfasalazine-induced renal injury have been reported [2,5,7,36]. Sulfasalazine-induced renal injury might lead to irreversible renal failure, organ transplantation, or even patient death [37–39]. There is no precise mechanism(s) for sulfasalazine-induced renal injury.

Some investigations indicated the involvement of oxidative stress and its consequent events in this complication [8,9]. Oxidative stress and its consequences also seem to be involved in other sulfasalazine side effects including infertility and hepatic injury [40–42]. In line with previous investigations, we found that sulfasalazine significantly increased oxidative stress biomarkers in the kidney tissue (Table 1). Oxidative stress constitutes an important risk factor for tissue damage and organ dysfunction. Furthermore, an important interplay exists between oxidative stress and mitochondrial function [43]. Oxidative stress is involved in the activation of several signaling pathways leading to the activation of transcription factors, gene expression and induction of apoptosis [44]. Sulfasalazine-induced oxidative stress might contribute to mitochondrial injury induced by this drug. On the other hand, oxidative stress might be a cause or a consequence of mitochondrial dysfunction [16]. Therefore, sulfasalazine-induced ROS formation might deteriorate mitochondrial injury and vice versa.

High and constant dependence of kidney proximal tubular cells on energy mentions the critical role of proper mitochondrial function in this organ [13,14]. Sulfasalazine-induced mitochondrial dysfunction might lead to the energy crisis, tubular cells injury, and defect in ion and electrolytes reabsorption. The electrolyte imbalance in case reports of sulfasalazine or mesalazine [45–48] might be attributed to insufficient ion reabsorption (an energy dependent process) in the kidney.

As mentioned, sulfasalazine is metabolized to mesalazine and sulfapyridine by bacterial azoreductase enzymes in the human intestine. The contribution of the whole molecule of sulfasalazine and/or its each intestinal metabolite in the renal injury and oxidative stress induced by this drug is not clear. Some investigations mentioned that the obstructive nephropathy induced by sulfasalazine might be associated with sulfonamide crystals in the kidney [39]. It has also been found that sulfasalazine nephrotoxicity might be attributed to the intratubular precipitation of sulfapyridine crystals [3]. Hence, sulfapyridine-induced renal injury might play a role in sulfasalazine-induced renal injury and mitochondrial dysfunction. On the other hand, mesalazine (mesalamine) itself is widely administered against inflammatory bowel disease and rheumatoid arthritis [49]. Mesalazine administration is also associated with renal injury [46,50]. The mechanism of mesalazine nephrotoxicity is unknown, but it is presumed to be similar to that of other salicylates. Salicylates nephrotoxicity is associated with renal hypoxia, mitochondrial injury, uncoupling of oxidative phosphorylation, and inhibition of renal prostaglandin synthesis [51,52]. Hence, a part of sulfasalazine-induced mitochondrial injury in the current investigation might be attributed to the mesalamine as an intestinal metabolite of sulfasalazine.

The exact mechanism/effect of sulfasalazine-induced renal injury needs further investigations to be precisely cleared. It is not determined whether sulfasalazine and/or its metabolites are responsible for oxidative stress, mitochondrial dysfunction and renal injury induced by this drug. In the current investigation, we found that sulfasalazine administration caused mitochondrial dysfunction in rat kidney. However, the contribution of sulfasalazine and each of its intestinal metabolites on kidney mitochondria could not be drawn from these data. On the other hand, we found that different concentrations of sulfasalazine caused toxicity in isolated kidney mitochondria (data not shown). However, we are not able to rule out the role of sulfasalazine metabolites in kidney mitochondrial injury.

More investigations are needed to reveal the clinical significance of mitochondrial dysfunction and oxidative stress in the pathogenesis of sulfasalazine-induced renal injury. Moreover, the role of each metabolite of sulfasalazine and their effect on kidney mitochondria in different experimental models could be the subject of future investigations.
