# Expression of Concern: Sulfasalazine induces mitochondrial dysfunction and renal injury

**DOI:** 10.1080/0886022X.2026.2666991

**Published:** 2026-05-06

Publish as Expression of Concern:

We, the Publisher and Editor of *Renal Failure*, are issuing an Expression of Concern for the following article:

Niknahad, H., Heidari, R., Mohammadzadeh, R., Ommati, M. M., Khodaei, F., Azarpira, N., … Najibi, A. (2017). Sulfasalazine induces mitochondrial dysfunction and renal injury. *Renal Failure*, *39*(1), 745–753. https://doi.org/10.1080/0886022X.2017.1399908

Following publication of this article, concerns about the integrity of Figure 3C in this article and Figure 5D in author’s previous publication were brought to the Publisher’s and Editor’s attention:

Heidari R, Rasti M, Shirazi Yeganeh B, Niknahad H, Saeedi A, Najibi A. Sulfasalazine-induced renal and hepatic injury in rats and the protective role of taurine. Bioimpacts. 2016;6(1):3-8. doi: 10.15171/bi.2016.01. Epub 2016 Mar 28. PMID: 27340618; PMCID: PMC4916549.

As part of the investigation, the authors were asked for any information that would confirm the validity of Figure 3C. The authors state that they cannot access the original image. As we continue to work through the concerns raised regarding Figure 3C, we advise readers to interpret the information presented in the article with due caution.

The authors have been notified about this Expression of Concern.

